# Participatory action research to align Health and Demographic Surveillance System (HDSS) priorities with community needs in Uttar Pradesh, India

**DOI:** 10.1017/S1463423626101157

**Published:** 2026-03-31

**Authors:** Shalini Singh, Raghukul Ratan Pandey, U. Venkatesh, Ram Shankar Rath, Anil Ramesh Koparkar, Pradip Kharya, Hariom Pandey, Neetisha Besra, Hari Shanker Joshi, Anand Mohan Dixit, Vikasendu Agarwal, Brian Wahl

**Affiliations:** 1 Johns Hopkins India, India; 2 Department of Community and Family Medicine, All India Institute of Medical Science, Gorakhpur, India; 3 Department of Medical Health and Family Welfare, Government of Uttar Pradesh, India; 4 Indian Council of Medical Research, Regional Medical Research Centre, Gorakhpur, India; 5 Department of Epidemiology of Microbial Diseases, Yale School of Public Health, USA

**Keywords:** Civic science, community engagement, Health and Demographic Surveillance System, participatory research

## Abstract

**Background:**

Participatory action research (PAR) methods effectively engage communities in identifying health priorities in low-resource settings. This study applied PAR methods during the establishment of a Health and Demographic Surveillance System (HDSS) in Gorakhpur, Uttar Pradesh, India.

**Methods:**

We implemented seven PAR methods across rural and urban populations: transect walks, resource mapping, social mapping, seasonal mapping, focus group discussions, semi-structured interviews, and community ranking exercises. Data were collected in September 2023 following facilitator training. Visual materials and field notes were thematically categorized, while audio-recorded interviews were transcribed, translated, and analysed with combined inductive-deductive thematic analysis. Community health priorities were quantitatively analysed through free listing, pile sorting, and ranking.

**Results:**

Five major themes emerged: (1) Community context revealed diverse caste compositions and essential infrastructure; (2) Health priorities included acute conditions (fever, joint pain), chronic diseases (hypertension, diabetes), healthcare access limitations, and WASH issues; (3) Key contributors to poor health encompassed inadequate WASH facilities, limited health awareness, and discrimination; (4) Healthcare access barriers included absent primary care facilities, financial constraints, limited transportation, and poor service quality; (5) Community suggestions addressed both social determinants and healthcare services, emphasizing governance participation, WASH interventions, and improved healthcare infrastructure.

**Conclusions:**

PAR methods successfully identified community-defined health needs and barriers, demonstrating consistency across urban and rural settings. Community members provided concrete recommendations for health system improvements, validating the importance of incorporating local voices in HDSS design. The alignment between community priorities and documented regional health challenges underscores how participatory methods can shape surveillance activities to foster sustainable health improvements and enhance governance capacities. These insights directly informed HDSS operational plans, creating a foundation for responsive and equitable health surveillance systems.

## Introduction

The Health and Demographic Surveillance System (HDSS) is a longitudinal data collection platform for systematically gathering demographic and health data from a defined population (Sankoh and Byass, 2012; Herbst et al., 2021). HDSS sites collect household- and individual-level data at specified intervals and monitor vital events (e.g., births, deaths, migration) as they occur. HDSS sites support core public health functions such as surveillance, policy evaluation, and research activities (Martin-Moreno et al., 2016; Bhandari et al., 2020). HDSS platforms are particularly valuable for generating locally relevant evidence, where routine health data systems are often incomplete or unreliable (Herbst et al., 2021). Regarding research, HDSS sites can provide valuable sampling frames for targeted anthropological, biomedical, and demographic research (Dewi et al., 2018). Additionally, they offer valuable training opportunities for health personnel (Ng et al., 2009; Ghosh et al., 2015).

Engaging the community in HDSS activities ensures that research priorities reflect community perspectives and priorities (Sharma et al., 2023). The World Health Organization’s (WHO) framework for meaningful community engagement emphasizes that such approaches are particularly critical in marginalized populations, where health disparities are greatest (World Health Organization, 2016). Meaningful and intentional community engagement activities can advance social justice and equity by enabling health research within underserved communities (Adsul et al., 2024). Further, community perspectives can influence every stage of the research process, from conception to dissemination (Pratt, 2021a).

Several studies have explored aspects of community engagement activities at HDSS sites, including potential barriers, ethical considerations, and proposed models (Twine et al., 2016; Cunningham et al., 2019; Adedini et al., 2021; Hinga et al., 2021; Sharma et al., 2023; Yadav et al., 2023). However, evidence from HDSS sites on community engagement has focused on the effectiveness of community engagement strategies and how community engagement can support HDSS research activities (Sharma et al., 2023). Limited documentation remains on how participatory research methods can be effectively implemented as a systematic approach to community engagement (Wariri et al., 2017). Participatory research methods, including participatory action research (PAR), involve community members as active collaborators rather than passive subjects in research. This enables them to contribute meaningfully to identifying health priorities, designing research questions, and interpreting findings (Cornish et al., 2023).

In this context, we aimed to use PAR methods to guide the establishment of an HDSS in Gorakhpur, India. By centring community voices during the HDSS design phase, the study aimed to: (1) identify community-defined health needs and priorities, (2) understand perceived barriers to healthcare access, and (3) co-develop a foundation for sustained community engagement. This work addresses a critical gap in the literature by demonstrating how participatory methods can move beyond tokenistic engagement to build more responsive and equitable surveillance systems, recognizing that meaningful participation requires ongoing negotiation of power dynamics between researchers and community members (Chung and Lounsbury, 2006).

## Materials and methods

The present study draws on the civic science framework, which aims to enhance public participation in scientific knowledge creation and application (Bäckstrand, 2003; Twine et al., 2016). This framework aligns with a community engagement continuum that recognizes the progression from minimal community involvement to community-led research, shaped by contextual factors including trust, relationship building, and transparency (Key et al., 2019). Ethical approval was obtained from the Institutional Ethics Committee of the All India Institute of Medical Sciences (AIIMS) Gorakhpur (Approval No. IHEC/AIIMS-GKP/BMR/197/2023).

We used PAR methods to engage community members during the establishment of the site. Specific activities included community mapping, key informant interviews, focus group discussions (FGDs), and seasonal calendars to enable local populations to share and analyse their knowledge of life experiences (Cornish et al., 2023). Participants for FGDs and interviews were purposively sampled to ensure representation across gender, age, caste, and socio-economic strata. Selection was facilitated by local field workers based on their familiarity with the community and its informal leadership structures. While many participatory techniques used in this study, such as transect walks and social mapping, originated in participatory rural appraisal (PRA) traditions (Chambers, 1994), we employed them within a broader PAR framework.

### SEWARTH HDSS and study setting

SEWARTH (Supporting Equitable Wellness through Advanced Research and Training in Health) is a collaborative initiative between the Uttar Pradesh Department of Medical Health and Family Welfare, the AIIMS Gorakhpur, the Johns Hopkins Bloomberg School of Public Health, and the Yale School of Public Health. The partnership aims to establish a centre of public health excellence in Uttar Pradesh by developing a dedicated HDSS for public health education, training, and research. Recognizing the importance of addressing power asymmetries in global health research (Pratt, 2021b; Bayingana et al., 2025), we intentionally assembled a research team that embedded local knowledge throughout the data collection process. All authors were based in India during the study period.

Gorakhpur is located in eastern Uttar Pradesh, a region where health services are constrained by uneven facility distribution, inadequate rural transportation, limited awareness, and health workforce challenges (Ghosh, 2017; National Health Mission, 2023; Pandey and Chaturvedi, 2023). The district experiences poor health and nutrition outcomes among children less than 5 years and women of reproductive age. The infant mortality rate in 2019–2021 in Gorakhpur district was 37 per 1,000 live births (International Institute for Population Sciences, 2021), and the maternal mortality ratio in 2017–2019 was 272 per 100,000 live births (Goli et al., 2022). The HDSS comprises both rural and urban populations in Gorakhpur, with a distribution similar to the typical rural-urban distribution in Uttar Pradesh. Table 1 provides a comparison of relevant demographic and health indicators across India, Uttar Pradesh, and Gorakhpur District. Supplementary File 1 details population distribution by gender and social categories in villages covered by SEWARTH HDSS.

**Table 1.** Comparison of relevant national, Uttar Pradesh, and Gorakhpur District demographic and health indicators

	India	Uttar Pradesh	Gorakhpur District
Population (2021)	1,363,006,000*	232,301,000*	5,110,000^†^
Female literacy prevalence (2019–2021)	71.5%^‡^	66.1%^‡^	68.8^‡^
Sex ratio at birth (2021)	900*	888*	943^‡^
Infant mortality rate (2019–2021)	35.2^‡^	50.4^‡^	37.1^‡^
Under-5 mortality rate (2019–2021)	41.9^‡^	59.8^‡^	60.1 (2015–2016)^§^
Maternal mortality ratio (2020–2022)	88 (2020–2022)	141^#^ (2020–2022)	272 (2017–2019)
Life expectancy at birth (2013–2017)	69^∥^	65^§^	NA

* Population Projections for India and States (2011–2036); Report of the Technical Group on Population Projections (July 2020).

† Projections based on decadal growth rate

‡ National Family Health Survey-5 (2019–2021)

§ Bora JK, et al. Neonatal and under-five mortality rate in Indian districts with reference to Sustainable Development Goal 3: An analysis of the National Family Health Survey of India (NFHS), 2015–2016. PLoS One. 2018 Jul 30;13(7):e0201125.

∥ Goli S, et al. Estimates and correlates of district-level maternal mortality ratio in India. PLOS Glob Public Health. 2022 Jul 18;2(7):e0000441.

# Sample Registration System, Office of the Registrar General of India

### PAR process

**Figure 1.**
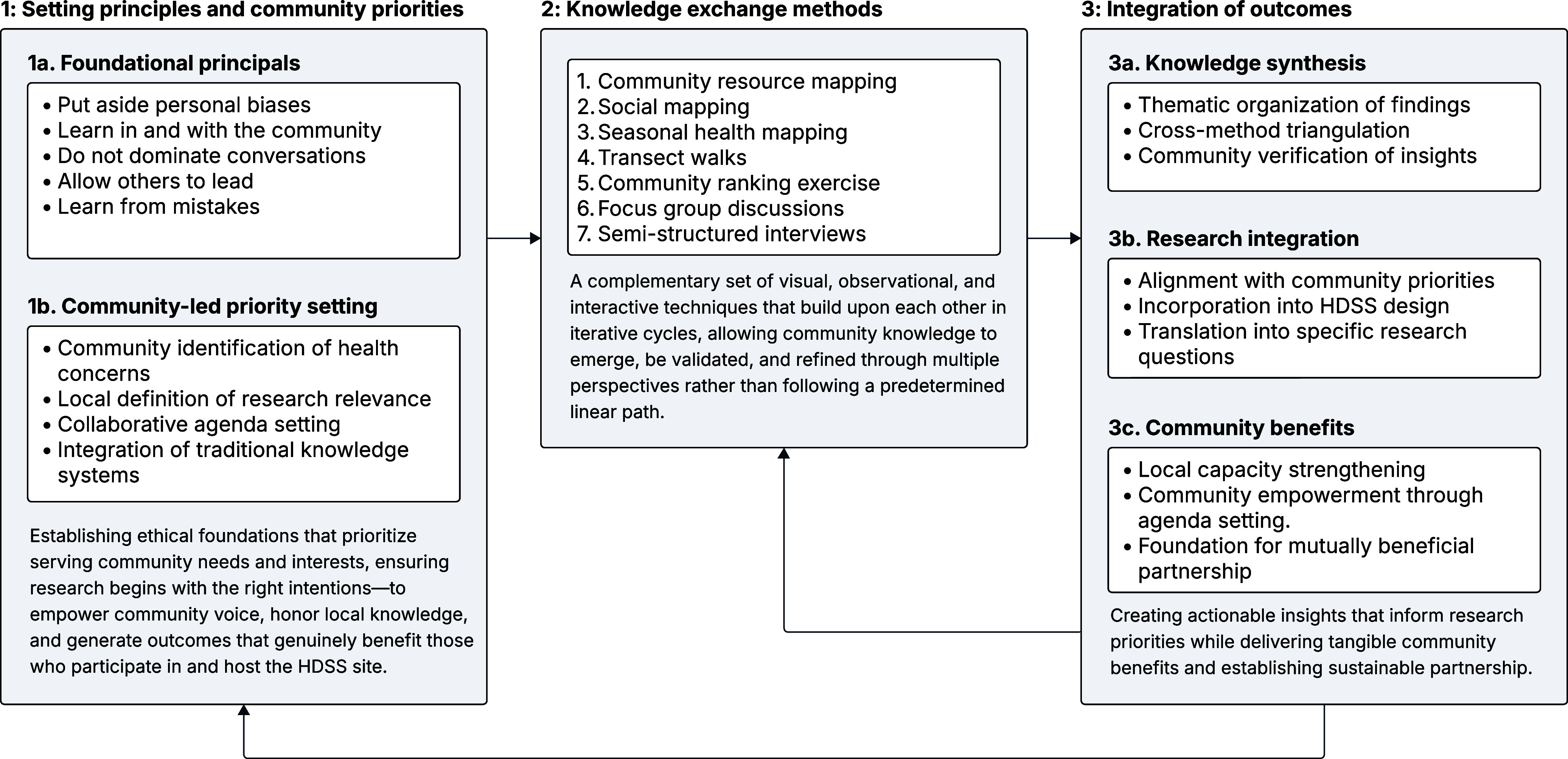
PAR process for community engagement. A three-step process, based on principles of civic science, for PAR within the context of establishing a health and demographic surveillance system site.

The PAR process followed a structured framework designed to centre community knowledge and participation (Figure 1). Seven complementary PAR methods were systematically implemented across all study sites, which include: community resource mapping, social mapping, seasonal health mapping, transect walks, community ranking exercises, FGDs, and in-depth interviews (IDIs) (Figure 1 and Table 2). These methods were implemented over a four-week period in September 2023. Each activity followed standardized protocols to ensure consistency while remaining responsive to local contexts. Operational definitions, participant composition, and implementation details are summarized in Table 2.

**Table 2.** Details of PAR methods used for CE in SEWARTH HDSS

Participatory method	Purpose	Process
Transect walk	To systematically observe, document, and analyse the community’s social assets, physical infrastructure, and environmental characteristics and gain a comprehensive understanding of available resources and the context in which they exist.	Conducted in each village/ward to observe and analyse social assets, infrastructure, and environment. Team of 10 members from AIIMS Gorakhpur/JHU, community representatives, and local community members ensuring diversity in age, gender, and socio-economic status. Routes were chosen with community input for maximum information. Active participation and input were ensured by community members. Walks mapped sites, resources, and social groups. Noted features, interacted with shopkeepers, and identified vulnerable households. Discussed prevalent seasonal diseases. Debriefing after the end of the walk summarized collective insights and learnings with the community.
Community resource mapping	To systematically identify and list the community’s resources, along with its underlying social infrastructures and networks, to provide a holistic understanding of the assets and relational dynamics within the community.	Conducted with 10–15 community representatives, women collective members, and active youth. Mapped various land uses, shops, health facilities, water sources, schools, Gram Panchayat Bhawan (local government office), drainage systems, community toilets, ASHA(CHWs) and Pradhan houses, alcohol shops, and temples. Facilitated discussions to gather additional information and identify challenges or opportunities. Highlighted patterns, connections, and relationships between resources and social structures.
Social mapping	To understand community dynamics, relative power structures (influential individuals and groups) and social networks, which can influence decision-making and resource distribution	Involved 10–12 residents from various religions, castes, and age groups. Mapped different social groups in the village and their interactions. Participants discussed and grouped related social structures. Identified potential relationships between resources and social structures.
Seasonal mapping	To analyse and document the cyclical patterns of disease prevalence throughout the year, providing insights into the temporal dynamics of health challenges in the community.	Engaged 10–12 active local individuals from the community, including both men and women. Encouraged identification of key health challenges and opportunities for each season. Participants mapped health risks and health seeking pattern by season.
Ranking by community	To understand the perceived priorities for improving health in the community.	Involved 15–18 residents both men and women, from different religion, caste and age-groups. First, everyone was asked about diseases and health priorities or problems. Listing of diseases and health problems on posters. Voting by each participant of the listed items. Scoring and priorities given to items with maximum scores.
Focus group discussions (FGDs)	To identify community strengths, common health challenges faced by the community, contributing factors at the community level, and gathering their collective suggestions for improving healthcare services.	12 FGDs one each in 11 villages and one urban ward covered within HDSS Each FGD involved 7–10 participants, comprising a mix of adult men and women, ranging from 19–67 years of age, and representing marginalized, vulnerable or low- income households. The participants meeting the above criteria, ready to spare time and could provide rich perspectives about the health and social context of their village were identified and recruited by the local facilitators for the FGD. Informed written consent was obtained from all the participants before commencing the FGD.
Semi-structured interviews with representatives from the local self-government (Pradhans and Parshad)	To understand the main healthcare facilities in the villages covered within the HDSS area, including their services, accessibility, and quality, identifying any barriers to healthcare access and health providers’ perceptions of disease prevalence. Additionally, the interview aimed to explore proactive measures for preventive healthcare, health awareness, and strategies for engaging the community in health initiatives.	11 Pradhans of the villages and One Parshad of the urban ward falling within the HDSS area were interviewed. These community representatives were briefed on the HDSS and PAR process beforehand during the orientation, after which local facilitators personally contacted them, obtained consent and undertook the interview.
Semi-structured interviews with senior citizens	To explore the participant’s individual health issues and their experiences with government health systems, accessing healthcare services, including any barriers they face such as affordability, distance, health providers behaviour or availability. Additionally, the interview sought to understand concerns related to elder abuse, neglect, or exploitation within the community.	Senior citizens were prioritized for their rich historical perspective on community health, trends, and traditional practices. As a vulnerable group, they can highlight gaps in the health system and barriers to care, informing potential solutions. Facilitators with community members in their team purposefully selected and contacted senior citizens over 60 from the community, choosing those who could provide detailed insights. They personally contacted 12 of them, obtained consent and undertook the interviews.

In August 2023, a three-day facilitator training workshop was conducted. Following the training, six diverse data collection teams were formed. Data collection was conducted by 35 trained facilitators (16 male, 19 female), of whom 28 were residents of Gorakhpur district. This team included community nursing students from AIIMS Gorakhpur, research associates from Deen Dayal Upadhyaya Gorakhpur University (DDUGU), and individuals from a local NGO, Manav Seva Sansthan, which has been active in the community for several decades. Data collectors reflected the religious and caste diversity of the study area, including Hindu (*n* = 29) and Muslim (*n* = 6) team members, and represented general caste (*n* = 11), ‘other backward class’ (*n* = 15), and scheduled caste (*n* = 9) categories. This diversity was intentionally maintained to ensure sensitivity to local social hierarchies and to facilitate inclusive participation during field activities.

Before implementing the PAR methods, we conducted a structured orientation process with local stakeholders. A half-day dialogue session was organized with village and ward heads from the proposed HDSS sites. This session introduced the HDSS concept and our community engagement plan. In addition, during this period, FGD and IDI guides were piloted with five individuals and refined based on their responses. The interview guides are available in the supplementary materials.

Prior to data collection, each participant was informed about the research aims and objectives. Informed consent was obtained from each of the selected participants and local leaders of the selected areas. No monetary inducements were provided; participants received only light refreshments. FGDs and IDIs were conducted in an open setting. All interviews were audio-recorded and supplemented with detailed field notes. Data were collected until saturation was reached. Recorded data were transcribed verbatim, avoiding interpretive additions. Regular team reflections after field activities examined how research team positionality and assumptions might have influenced data collection and interpretation.

### Data analysis

We analysed data using a multi-method approach to ensure rigorous interpretation of PAR findings. Visual materials and field notes from transect walks and mapping exercises were categorized thematically, while community health priorities were quantitatively analysed by free listing, pile sorting, and ranking. Audio-recorded interviews and discussions were transcribed verbatim in Hindi, translated into English, and uploaded into MaxQDA version 22. We applied a combined inductive-deductive thematic analysis approach. First, a preliminary coding framework was developed based on study objectives and existing literature. This included codes for social determinants of health, barriers to care, perceptions of healthcare quality, and community strengths. During iterative reading of transcripts, emergent codes were added to capture context-specific themes. Two researchers independently coded a subset of transcripts to ensure inter-coder consistency, and discrepancies were resolved through discussion. Findings from each method (e.g., FGDs, IDIs, ranking exercises) were then triangulated to validate thematic consistency. We also examined whether themes and priorities differed between urban and rural settings within the HDSS area. Preliminary results were shared in community feedback sessions where participants clarified and validated the interpretations.

## Results

We identified five broad themes during the PAR activities. First, we present the HDSS community context. Second, we detail the community’s perceived priorities for health improvement. Third, we explore key contributors to poor health mentioned by community members. Fourth, we examine barriers to healthcare access. Last, we present community suggestions for improving healthcare services. Supplementary File 2 provides participant profiles from focus groups and IDIs during the PAR process.

### Community context

**Figure 2.**
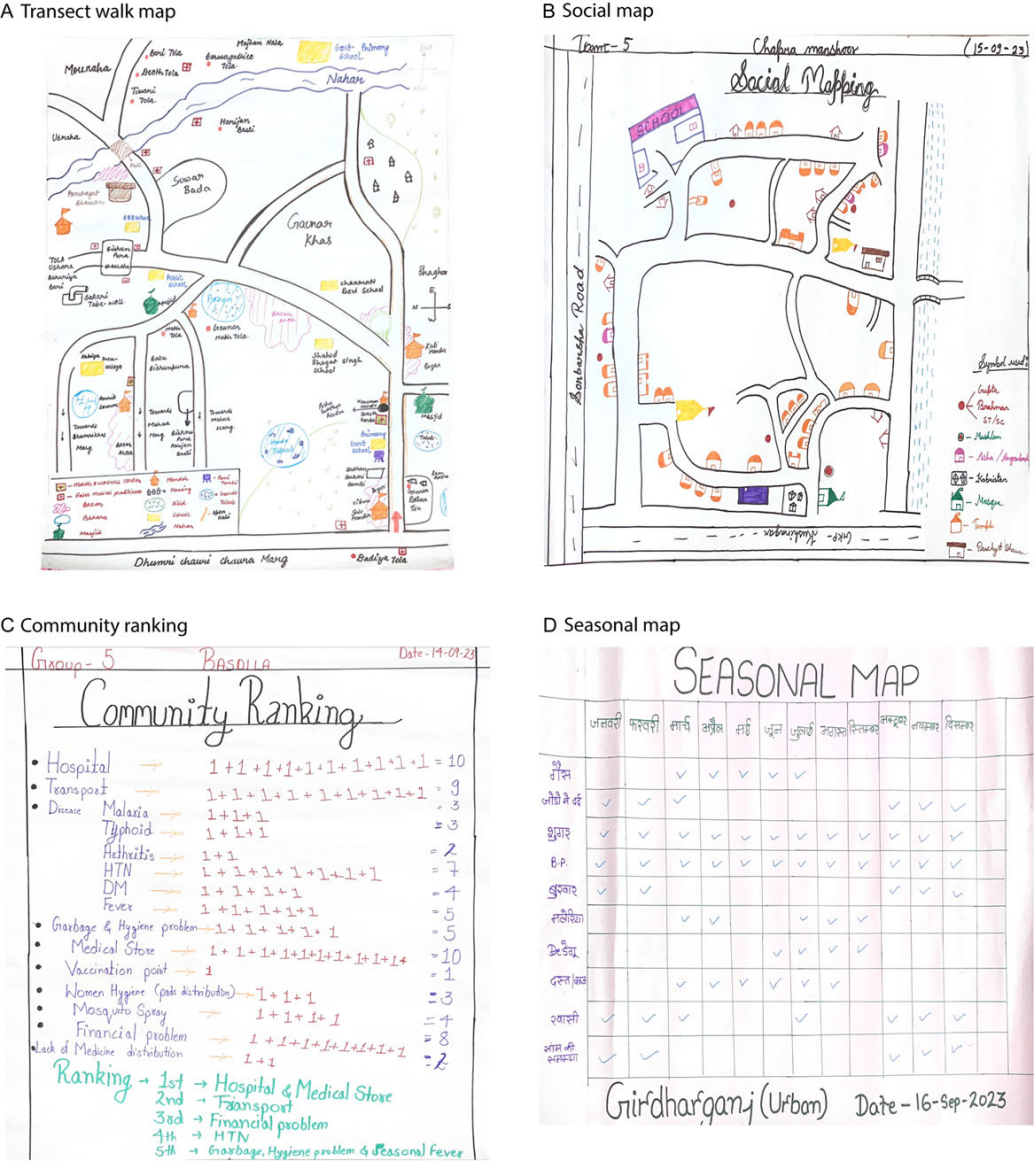
Outputs of the visual mapping and ranking activities. Outputs are illustrative examples from select villages. A. Map prepared following the transect walk undertaken in Village 9 to understand resources and physical infrastructure; B. Map prepared by community members from Village 9 to understand social dynamics; C. Seasonal map from Ward 25; D. Results from community ranking exercise in Village 5.

In Gorakhpur, as in much of northern India, caste identity remains a major social determinant of health and well-being. Most villages within the rural component of the HDSS area featured diverse caste compositions, including advantaged and oppressed castes and minority religious communities. Small tribal populations also reside in select villages. These communities typically reside in distinct hamlets called *bastis* and *tolas* (i.e., both small settlements within villages). Those from scheduled castes, which are a group of constitutionally recognized disadvantaged castes, reside in settlements that are generally located on the periphery of villages. This spatial and social stratification, which was less apparent in the urban component of the HDSS, influences patterns of healthcare utilization, exposure to environmental risks, and participation in governance processes. During participatory mapping exercises, community members identified these divisions as shaping both everyday interactions and opportunities to engage with formal health systems. Essential infrastructure identified through the resource mapping exercise included handpumps and water tanks, primary schools, local government offices, Anganwadi Centers (i.e., village-based government childcare and nutrition centres), and government primary care health facilities. Outputs of the mapping exercises and results of the ranking process undertaken from a sample of HDSS villages covered in the PAR exercise are included in Figure 2.

Participants highlighted several community strengths during FGDs, including cultural heritage, safety, social cohesion, and participatory governance. Many credited elected village-level local self-government institutions in rural India, called *Gram Panchayats,* for infrastructure development and improved access to essential services.

*People help each other, especially in difficult times. I think this is our biggest strength (Female 1, Village B, FGD 2).*

*The active participation of residents in local governance and decision-making processes is commendable. It creates a sense of ownership and responsibility towards our village (Male 8, Village K, FGD 11).*

*Our village is clustered around one of the oldest sugar mills in India. Due to this, we have many facilities, which one would not expect in other villages. We have banks, medical stores, and private doctors (Male 3, Village B, FGD 2).*

They also noted positive societal educational changes, particularly for girls.

*I am observing that now people are more serious about the education of their children, especially girl children. This is opposite to my time (Female 6, Village C, FGD 3).*

Regarding the positive aspects of healthcare, several community members noted that the government is working to enhance health services. A few participants highlighted the opening of primary care facilities in their village. Others praised the efforts of community health workers, including ASHA workers (i.e., Accredited Social Health Activist; trained female community health workers who serve as a link between the community and public health system) in delivering door-to-door services and addressing women’s health issues.

*Our local ASHA worker is vital to our healthcare. She provides essential tablets for malaria, filaria, and fever, offers dietary advice, and makes house visits. She monitors pregnant women, educates on health practices and vaccinations, and arranges ambulance services in emergencies (IDI-Female, 65 years, Village A).*

### Perceived priorities for improving community health

As part of the ranking exercise, we contacted approximately 130-160 participants from all 11 HDSS villages and one municipal ward, asking them to identify health-related concerns that should be prioritized to improve their health and well-being. Supplementary File 3 displays the cumulative frequencies for these priority health concerns in descending order.

Acute simple and routine health concerns ranked highest, including conditions such as fever (frequency: 79), joint pain (77), aches and pains (35), bloating (29), and itching (27). Chronic illnesses were also prominent; high blood pressure (38) and diabetes (33) were reported as common. Concerns about access to health services formed another significant category, including lack of health facilities (26), insufficient medical stores or pharmacies (24), inadequate health information (15), and limited transport options for seeking healthcare (9). Water, Sanitation and Hygiene (WASH) issues, such as garbage disposal challenges (23), unavailability of dustbins (5), and water drainage issues (9), indicated infrastructural deficiencies affecting public health. Financial barriers (21) related to accessing healthcare services suggested broader socio-economic issues affecting the community’s health and well-being. Health priority rankings showed similar patterns across urban and rural settings.

### Key contributors to poor health

Community members highlighted WASH-related issues as the most significant contributors to poor health, including inadequate drainage and sanitation facilities, lack of access to safe drinking water, limited vector control, and high levels of mosquito breeding.

*We have all been affected by dengue. The unclean surroundings, lack of drainage and stagnant water in our vicinity have led to an increase in mosquito breeding, exacerbating health issues (IDI-Male 65, Village E).*

Several community members expressed their concern about the lack of safe and clean drinking water in their village.

*There is a significant water problem in our village, causing inconvenience. Installation of piped water supply taps has been limited, and clean and safe drinking water availability is a big challenge (IDI-P, Village E).*

Participants attributed poor health in their communities to a lack of awareness about health services, rights, and entitlements, which was intertwined with social, cultural, and structural determinants of health.

*Some community members, especially from marginalized communities, are not fully aware of the healthcare services available or how to utilize them. This lack of awareness leads to underutilization of the services and can increase health issues in the long term (IDI-P, Village G).*

*In my opinion, those who belong to low socioeconomic groups often get sick because they do not know much about health services and lack information about staying healthy (IDI-P, Village E).*

Some community members described experiencing discrimination from health systems based on geographic or social identity. Rural community members felt marginalized and devalued compared to urban residents when seeking care. This sense of alienation and fear of unfair treatment leads them to avoid formal healthcare services.

*City hospitals discriminate between urban and rural people… so we avoid seeking care (Male-9, Village F, FGD 6).*

Financial barriers and mistrust in public health facilities also contributed to the community’s preference for alternative healthcare options.

*Money drives the quality of care you would receive. Most of the people in our village are very poor. They cannot afford to become ill. People go to quacks (informal providers) as the first line of treatment and that is the reason even minor illnesses progress into fatal diseases (Male-5, Village C, FGD 3).*

Health determinants were consistently identified across the urban ward and the rural villages.

### Barriers to accessing health care services

While initial community responses often focused on immediate healthcare access needs, deeper engagement through iterative FGDs and ranking exercises revealed underlying structural and social factors shaping health outcomes. These barriers reflect the concerns that emerged as participants moved beyond immediate priorities to discuss root causes.

The HDSS community identified four primary barriers to accessing health care: (1) absence of primary care facilities, (2) financial constraints, (3) limited transportation options, and (4) poor quality of primary health care services – see Table 3. These barriers highlight the complex challenges associated with primary health care delivery in rural Gorakhpur.

**Table 3.** Perceived barriers to accessing health care services by the HDSS community

Types of barriers reported	Experience of the community members
Lack of health facilities	*In our village, one of the major problems is that we do not have a hospital close by. If we had a hospital right here in our village, it would be much easier for everyone to get treatment quickly (IDI-P, Village H).* *The primary health center is quite far, causing difficulties in reaching it during emergencies. (Male 8. 58 years, Village D FGD-4)* *The hospital is located far away from our village, which presents challenges, particularly in obtaining medicine during the night. We often rely on ASHA and Anganwadi workers for support in addressing our health challenges (IDI-Male 57, Village K).*
Financial Barriers	*My husband has been living with diabetes for the past 20 years and has been receiving treatment from a private doctor in Gorakhpur city. Our ability to continue his medication is directly tied to our financial situation. When we have the funds, we purchase his medicine, but when money is tight, we are unfortunately forced to skip his medication. This is the reality of our lives. Currently, we are facing a financial crunch and are unable to afford the medicine (IDI-Female 60, Village H).* *Being financially constrained, I often have to resort to borrowing money from neighbors and relatives for medical treatment, especially in cases of major illnesses (IDI-Male 70, Ward 1).*
Lack of transport	*There are several primary barriers to accessing healthcare services in our village. One significant issue is the limitation in transportation. While we have ambulance services like 108 and 102, there have been instances of delays, especially critical during emergencies. This is a major concern for villagers living on the outskirts, who already face challenges due to the distance from healthcare facilities (IDI-P, Village F).* *The distance to the primary health center and the resulting transportation costs creates difficulties in accessing timely and affordable treatment (Male 2, 42 Years, Village D, FGD 4).*
Poor Quality Care	*A frequent issue we encounter is that many doctors prescribe medications that are only available in private pharmacy, which increases the cost significantly. They also often recommend diagnostic tests from private facilities. This leads us to question the benefit of visiting a government hospital if we still must bear high expenses (IDI-Female, 65 years, Village H).* *Although the health providers at PHC Sardar Nagar are skilled and dedicated, there are times when they are overwhelmed due to the large rural population they serve. This often results in longer waiting times and, at times, hurried consultations (IDI-P, Village B).* *The health sub-center offers limited services, faces resource constraints, serve only those living in central village, leaving those in remote areas with reduced access to essential healthcare services (IDI-P, Village F).* *Local health workers are not competent enough to deal with the emergency. They should be trained properly and regularly (Male 9. 20 years, Village D FGD-4)* *Where health services are available, the quality varies. Some doctors at the Primary Health Center treat patients well, but others are negligent and prescribe expensive medicines that are difficult for the community to afford (Male 4. 67 years, Village D FGD-4).*

While community members appreciated the support of community health workers, they noted that most villages in the densely populated HDSS area lack accessible primary care facilities. Existing facilities primarily serve central village areas, leaving remote locations with marginalized populations underserved. These facilities offer limited services, resulting in high patient volumes at distant primary and secondary care centres that struggle to provide quality care due to heavy caseloads.

*One significant issue is the limitation in transportation. While we have ambulance services like 108 and 102, there have been instances of delays, especially critical during emergencies (IDI-P, Village F).*

Financial constraints severely limit community access to necessary health care. Despite government facilities being intended to provide free treatment, many community members reported out-of-pocket expenses for medications due to shortages or prescriptions for outside medications.

*Our ability to continue his medication is directly tied to our financial situation. When we have the funds, we purchase his medicine, but when money is tight, we are unfortunately forced to skip his medication (IDI-Female 60, Village H).*

Several participants expressed other quality concerns, including inefficient emergency referral transport, limited frontline workers’ skills in handling health emergencies, and negligent care by health providers at government-run basic healthcare facilities known as primary health centres (PHCs).

*Although the health providers at PHC Sardar Nagar are skilled and dedicated, there are times when they are overwhelmed due to the large rural population they serve. This often results in longer waiting times and, at times, hurried consultations (IDI-P, Village B).*

### Community’s suggestions to improve health and well-being

The HDSS community members offered suggestions for health improvement that addressed both root causes of poor health, including social determinants of health, and barriers to quality healthcare access.

For social determinants of health, community members stressed the need for inclusion in governance and health planning processes. They called for community participation in decisions, with emphasis on women’s involvement in decision forums. Participants requested better information about health rights and entitlements, especially for marginalized groups. These upstream concerns tended to emerge during later stages of FGDs and through ranking exercises, after initial discussions had focused on immediate service needs. Economic factors were mentioned through requests for jobs and poverty reduction programmes to address financial barriers. Community members stressed WASH interventions, including garbage removal, drain cleaning, and piped water supply. Participants requested supervision of staff who implement WASH activities and community ownership of these efforts.

For healthcare services, participants wanted more government hospitals and primary care facilities in villages to improve access. They requested emergency transport systems and respectful healthcare providers. Financial protection through health insurance and access to medicines were suggested to reduce expenses. Community members recommended health camps to increase service availability, along with training for frontline workers in emergency care. They also requested health education and nutrition programmes to address knowledge gaps.

## Discussion

This study demonstrates the feasibility and value of using PAR methods to understand the study area through community perspectives and lays the groundwork for ongoing engagement at a new HDSS site. The PAR process revealed complex relationships between social determinants of health, healthcare system limitations, and community priorities that might have remained hidden through conventional research approaches.

Our findings revealed several community-identified health priorities, including acute conditions, chronic diseases, healthcare access limitations, improved WASH, and financial barriers to quality care. These identified priorities align closely with documented health challenges in Uttar Pradesh (Dandona et al., 2017). Community members offered concrete suggestions addressing both root causes and access barriers, demonstrating their capacity to contribute meaningfully to health system planning. This alignment between community perspectives and state-level data validates the importance of incorporating local voices in health policy implementation. Our findings also converge with other health needs assessments in the region. Researchers identified gaps in actionable health information across the health system in Uttar Pradesh, which echoed the calls from the current study for better information about entitlements and health rights (Kapadia-Kundu et al., 2012). More broadly, community health needs assessments have been recognized as critical tools for identifying and prioritizing local health demands, targeting resources to address inequalities, and involving local people in health planning (Mathias et al., 2015; Chavan et al., 2018). However, while priorities for clinical services are consistently reported across such assessments, concerns about governance participation, health rights, and women’s inclusion in decision-making are less commonly elicited. The iterative and sustained nature of our PAR approach may have created space for these upstream concerns to surface.

The SEWARTH HDSS community demonstrated key strengths, including social cohesion, active local governance, and established community consultation practices. These assets provide a foundation for community-driven public health initiatives. The active role of *Gram Panchayats* (i.e., elected village-level local self-government institutions) in the PAR process underscores how existing governance structures can facilitate the triangulation of community voices with government priorities (Ministry of Health and Family Welfare, 2017).

We identified socio-spatial health inequities where disadvantaged communities cluster on village peripheries, with rural primary care facilities failing to integrate them effectively – patterns documented elsewhere in Uttar Pradesh (Blanchard et al., 2024). Community members also identified WASH-related issues as primary contributors to poor health, consistent with regional data showing persistent sanitation challenges and vector-borne disease risks in Gorakhpur (International Institute for Population Sciences, 2021; Paulraj et al., 2021). The four critical barriers identified – inadequate primary care facilities, financial constraints, limited transportation, and poor-quality care – reflect systemic challenges across Uttar Pradesh, where significant gaps exist in primary care infrastructure and households bear substantial out-of-pocket expenses (Choudhury et al., 2019; Ministry of Health and Family Welfare, 2024b). These structural challenges drive community reliance on informal providers, potentially worsening health outcomes for vulnerable populations (Singh et al., 2019).

While government initiatives like *Ayushman Bharat*, which aims to establish comprehensive primary care centres, and PM-JAY, which provides health insurance coverage to low-income households, aim to address many of the challenges identified by community members, implementation gaps persist. Gorakhpur’s relatively low PM-JAY registration rates (Ministry of Health and Family Welfare, 2024a) suggest barriers that future HDSS research should explore, particularly focusing on strengthening programme delivery for marginalized communities where healthcare needs are most acute.

Our study drew on the civic science framework (Bäckstrand, 2003) and community engagement continuum (Key et al., 2019) to shift decision-making power to community members. This approach facilitated bidirectional knowledge exchange, enabling researchers to gain local insights while community members actively shaped the research agenda. The PAR methods effectively triangulated community perspectives, government health priorities, and research objectives, creating a foundation for responsive and relevant HDSS implementation.

Several factors contributed to successful PAR implementation. Engaging village and ward heads built trusted relationships and crucial entry points. Developing the engagement plan through open dialogues with community leaders ensured ownership from the outset. Recruiting facilitators from local communities, NGOs, and educational institutions provided contextual understanding and cultural sensitivity. The plurality of methods – from visual mapping to ranking exercises – allowed for the triangulation of insights validated by local investigators, enhancing credibility. This initial engagement raised awareness about HDSS goals across several villages while addressing concerns about extractive research approaches often reported in surveillance settings.

An important finding was the consistency in health challenges and barriers between urban and rural areas within our HDSS site, suggesting systemic issues in primary healthcare across Gorakhpur. This similarity also likely stems from common challenges in social and environmental determinants affecting both settings.

The framing of participatory exercises can shape the priorities communities initially express, with perceived needs often focusing on access to clinical services before deeper engagement reveals underlying concerns (Mathias et al., 2015). Our use of multiple complementary PAR methods, including iterative discussions, ranking exercises, and seasonal mapping, was intended to move beyond initial responses and surface the structural and social factors shaping health in this community. We observed this pattern in our own data – while immediate healthcare access dominated initial responses, concerns about participation in governance, health rights, and women’s inclusion emerged through sustained engagement.

Key limitations include the potential for social desirability bias in group discussions and the challenge of capturing all community voices. Despite intentional diversity in our facilitation team, PAR processes can reflect dominant cultural norms; caste and gender hierarchies may have shaped which voices were heard in group settings and what concerns participants felt comfortable raising. Gender norms in Uttar Pradesh limited some women’s autonomous participation, though we mitigated this by engaging women through women-focused groups and individual interviews. Regular team reflections helped identify these dynamics, and recruiting facilitators from diverse caste and religious backgrounds may have enabled more inclusive participation than a homogeneous team would have achieved. Health priorities expressed in lay terminology may reflect recent visible challenges while overlooking stigmatized conditions. Future HDSS surveillance activities will complement these initial insights by systematically collecting data.

The PAR insights will inform HDSS implementation through three approaches. First, capacity-building workshops for AIIMS Gorakhpur students, faculty, and researchers will develop strategies addressing community priorities. Our findings will sensitize healthcare providers to community perspectives through the HDSS’s role as a research platform and training site. Second, findings will guide longitudinal survey design to track healthcare utilization patterns, access barriers, provider preferences, financial constraints, and WASH concerns. Third, we will use locally relevant evidence to strengthen programme and policy implementation, particularly addressing gaps in government initiatives.

This study demonstrates that embedding participatory methods at the foundation of HDSS development strengthens scientific relevance while building community trust and ownership. Community-identified priorities are now informing the health indicators tracked by the HDSS, and findings have been shared with the state health department to support local health planning. We have recruited community members as data collectors and are establishing a community advisory board comprising residents from diverse backgrounds to guide ongoing HDSS activities. The alignment between community-identified priorities and government health challenges underscores the importance of incorporating local voices in health system design. As the SEWARTH HDSS evolves, we plan to explore research questions jointly with the community and continue engagement to understand how priorities change over time. These community-grounded insights provide a path forward for implementing equitable, sustainable, and context-sensitive health policies that reflect the needs and solutions identified by the communities themselves.

## Supporting information

Singh et al. supplementary material 1 Singh et al. supplementary material

Singh et al. supplementary material 2 Singh et al. supplementary material

Singh et al. supplementary material 3 Singh et al. supplementary material

## Data Availability

Data without personal identifying data are available upon request (Dr. Anand Mohan Dixit; dnana2791@gmail.com).

## Supplementary material

To view supplementary material for this article, please visit https://doi.org/10.1017/S1463423626101157.
