# Supplementary material for: Participatory action research to align Health and Demographic Surveillance System (HDSS) priorities with community needs in Uttar Pradesh, India
Source: Prim Health Care Res Dev. 2026 Mar 31;27:e46. doi: 10.1017/S1463423626101157 (PMC13080540; doi:10.1017/S1463423626101157)
Supplement: Singh et al. supplementary material 2 — Singh et al. supplementary material [file S1463423626101157sup002.docx]

**Supplementary File 2 Participants Profile**

1. *FGD participants’ profile*

| **FGD Number** | **Total Participants** | **Male** | **Female** | **Age group range** |
| --- | --- | --- | --- | --- |
| FGD 1: Village-A | 8 | 4 | 4 | 19-56 |
| FGD 2: Village-B | 7 | 2 | 5 | 30-62 |
| FGD 3: Village-C | 9 | 2 | 7 | 19-62 |
| FGD 4: Village-D | 10 | 7 | 3 | 20-67 |
| FGD 5: Village-E | 8 | 3 | 5 | 24-57 |
| FGD 6: Village-F | 9 | 3 | 6 | 21-45 |
| FGD 7: Village-G | 7 | 3 | 4 | 32-55 |
| FGD 8: Village -H | 8 | 4 | 4 | 27-64 |
| FGD 9: Village -I | 8 | 3 | 5 | 40-65 |
| FGD 10: Village-J | 8 | 3 | 5 | 40-65 |
| FGD 11: Village-K | 8 | 4 | 4 | 34-65 |
| FGD 12: Ward-L | 10 | 6 | 4 | 20-67 |
| Total | **100** | **44** | **56** | 19 - 67 |

1. *In-depth Interviews participants’ profile*

| **Community Representatives** | **Gender (Male/Female)** | **Age** |
| --- | --- | --- |
| Participant 1: Village A | Male (Pradhan) | 44 |
| Participant 2: Village B | Male (Husband of Pradhan) | 41 |
| Participant 3: Village C | Male (Pradhan) | 39 |
| Participant 4: Village D | Male (Pradhan) | 46 |
| Participant 5: Village E | Male (Pradhan) | 47 |
| Participant 6: Village F | Male (Father of Pradhan) | 52 |
| Participant 7: Village G | Male (Husband of Pradhan) | 32 |
| Participant 8: Village H | Male (Pradhan) | 55 |
| Participant 9: Village I | Male (Pradhan) | 54 |
| Participant 10: Village J | Male (Pradhan) | 35 |
| Participant 11: Village K | Male (Assistant of Pradhan) | 38 |
| Participant 12: Ward L | Male (Corporator) | 40 |
| **Senior citizens** |  |  |
| Participant 1: Village A | Female | 65 |
| Participant 2: Village B | Male | 70 |
| Participant 3: Village C | Male | 62 |
| Participant 4: Village D | Male | 62 |
| Participant 5: Village E | Male | 65 |
| Participant 6: Village F | Male | 65 |
| Participant 7: Village G | Female | 65 |
| Participant 8: Village H | Female | 60 |
| Participant 9: Village I | Male | 70 |
| Participant 10: Village J | Female | 67 |
| Participant 11: Village K | Male | 70 |
| Participant 12: Ward L | Male | 70 |
