# Supplementary material for: Participatory action research to align Health and Demographic Surveillance System (HDSS) priorities with community needs in Uttar Pradesh, India
Source: Prim Health Care Res Dev. 2026 Mar 31;27:e46. doi: 10.1017/S1463423626101157 (PMC13080540; doi:10.1017/S1463423626101157)
Supplement: Singh et al. supplementary material 3 — Singh et al. supplementary material [file S1463423626101157sup003.docx]

*Supplementary File 3 Ranking of Community Health Priorities*

| Health or health related concerns | Cumulative frequency |
| --- | --- |
| Fever | 79 |
| Joint Pain | 77 |
| High Blood Pressure | 38 |
| Aches and pains | 35 |
| Diabetes | 33 |
| Bloating and stomach related issues | 29 |
| Itching | 27 |
| Skin diseases | 27 |
| Lack of health facilities | 26 |
| Typhoid | 25 |
| Lack of pharmacy | 24 |
| Garbage accumulation and lack of removal | 23 |
| Financial barriers for seeking care | 21 |
| Thyroid disorder | 19 |
| Eye problems | 17 |
| Lack of health information | 15 |
| Drainage issues | 9 |
| Limited transport to avail health care | 9 |
| Malaria | 7 |
| Unavailability of dustbin for garbage collection | 5 |
| Lack of doctors | 4 |
| Women hygiene-related issues | 3 |
| Loss of appetite | 2 |
| Breathlessness | 1 |
| Leucorrhea | 1 |
| Unclean toilets | 1 |
